# HOW DO OLDER AFRICAN AMERICAN COUPLES TAKE CARE OF EACH OTHER? A DYADIC, QUALITATIVE ANALYSIS

**DOI:** 10.1093/geroni/igac059.1153

**Published:** 2022-12-20

**Authors:** Amy Rauer, Wendy McClean Cooke, Lyndsey Hornbuckle

**Affiliations:** University of Tennessee Knoxville, Knoxville, Tennessee, United States; University of Tennessee, Knoxville, Tennessee, United States; University of Tennessee, Knoxville, Tennessee, United States

## Abstract

Guided by Lyons and Lee’s (2018) Dyadic Theory of Illness Management, the current study explored covariation in dyadic health management behaviors within a sample of seventeen older African American married couples (Mage=61.68 years; Mmaritalduration=32.29 years). We also examined if spouses agreed on how they took care of each other’s health and whether these patterns changed after couples participated in a health-focused intervention together. Prior to beginning a 12-week walking plus resistance training exercise intervention, spouses completed questionnaires asking them to list the common things they did to help take care of their partner’s health as well as what their partner did for them. Thirteen couples completed these questionnaires post-intervention. Data from both waves were analyzed using thematic analysis (Braun & Clarke, 2006). Five health management behaviors domains were identified (diet, exercise, self-care, medical compliance, relationship maintenance). Although both partners reported encouraging healthier diets and exercise, wives also reported promoting other health management behaviors. Couples had greater congruence in their appraisals of wives’ health management behaviors compared to husbands’, as wives recognized many things husbands did to take care of them that husbands did not report themselves. Patterns appeared stable over time. Findings suggest the incongruence in couples’ health management behaviors represented complementary efforts to support each other and revealed that husbands may be underestimating how much care they are providing to their wives. A promising method for addressing health disparities in this population may involve capitalizing on this clear investment that older African American couples have in each other’s health.

